# Precise Delivery of Nitric Oxide Controlled by Bioorthogonal Endocellulase Ameliorates Hindlimb Ischemia

**DOI:** 10.3390/bioengineering13020128

**Published:** 2026-01-23

**Authors:** Yating Zhang, Meng Qian, Ruowen Chu, Shengyu Li, Jiawen Yuan, Jian Zhao, Zhixin Xu, Mengmeng Xing, Huan Jiang, Bo He, Chao Chai, Guangyu Yang, Sen Yang, Yongzhen Wei, Qiang Zhao

**Affiliations:** 1 State Key Laboratory of Medicinal Chemical Biology, Key Laboratory of Bioactive Materials (Ministry of Education), Frontiers Science Center for Cell Responses, College of Life Sciences, Nankai University, Tianjin 300071, China; ytzhang1108@163.com (Y.Z.);; 2 Department of Vascular Surgery, Tianjin First Central Hospital, Nankai University, Tianjin 300192, China; 3 State Key Laboratory of Vascular Homeostasis and Remodeling, Health Science Center, The Institute of Cardiovascular Sciences, School of Basic Medical Sciences, Peking University, Beijing 100191, China; 4 State Key Laboratory of Microbial Metabolism, Joint International Research Laboratory of Metabolic & Developmental Sciences, School of Life Sciences and Biotechnology, Shanghai Jiao Tong University, Shanghai 200240, China; 5 Department of Radiology, Tianjin Institute of Imaging Medicine, Tianjin First Central Hospital, School of Medicine, Nankai University, Tianjin 300192, China

**Keywords:** nitric oxide, targeted delivery, hindlimb ischemia, enzyme–prodrug therapy (EPT), endocellulase

## Abstract

Peripheral artery disease (PAD) remains a great threat to the health of older people globally. Nitric oxide (NO), as an important signaling molecule, is integral to processes such as angiogenesis, inflammation, and tissue regeneration, making it a potential candidate for PAD treatment. Nevertheless, NO—based therapies are frequently limited in clinical utility, primarily due to the lack of effective strategies for fine-tuning the release of exogenous NO. In this study, we developed an enzyme—prodrug pair based on endocellulase (Cel5A-h38), which ensured complete bioorthogonality, thus avoiding interference with endogenous enzymes and eliciting an inflammatory response. This delivery system enables localized and controlled NO release, thus preventing side effects induced by systemic exposure. The therapeutic efficacy of the NO delivery system was systematically evaluated in a porcine model of hindlimb ischemia. Our results confirmed the benefits of targeted NO delivery in hindlimb ischemia, which include enhanced neovascularization and tissue perfusion, reduced inflammation, and alleviated muscle fibrosis, demonstrating its optimal translational potential.

## 1. Introduction

Peripheral artery disease (PAD), a condition affecting over 200 million people worldwide [1,2], is caused by atherosclerotic arterial narrowing and leads to progressive limb symptoms including claudication, rest pain, and skeletal muscle myopathy, with advanced stages culminating in critical limb-threatening ischemia (CLTI) [3,4]. Although large-artery revascularization, such as bypass surgery or stent implantation, remains the primary treatment for severe PAD, unfavorable anatomy or a high surgical risk often precludes its use in many patients [5,6,7]. Novel therapeutics for PAD are being pursued based on recent clinical findings, which indicate that simultaneously enhancing tissue perfusion and abrogating calf skeletal muscle abnormalities may have the most significant impact [8,9,10].

Nitric oxide (NO) was initially identified as a short-lived gaseous signaling molecule produced by endothelial nitric oxide synthase (eNOS) that plays an essential role in maintaining vascular homeostasis by regulating vascular tone, inhibiting platelet aggregation, preserving normal vascular permeability and modulating immune responses [11,12,13,14,15,16]. Efforts to directly augment NO bioavailability in PAD patients have produced mixed or modest clinical benefits, highlighting the fundamental limitations of systemic delivery [17,18,19]. These observations suggest that insufficient local NO bioavailability within ischemic muscle underlies the limited efficacy of prior NO-centered therapeutics in PAD. These barriers motivate strategies that impose spatial and temporal control over NO exposure. By enhancing targeting accuracy and dose control of NO, enhanced limb perfusion in hindlimb ischemia (HLI) models has been validated by us and other groups [20,21,22].

In our previous research, we constructed an NO delivery system through modification of an enzyme–prodrug pair of galactosidase–galactosyl–NONOate, employing a ‘bump-and-hole’ strategy. Despite these advancements, the system has not accomplished full bioorthogonality. The engineered mutant galactosidase (A4-β-Gal^H363A^, H363A) retains the ability to recognize and cleave endogenous galactosidic bonds. Additionally, the application of H363A may induce certain inflammatory responses due to the immunogenicity of galactosidase [20]. To address these issues, we recently developed a targeted delivery system in which NO generation is triggered by endocellulase, an enzyme that is absent in human cellular metabolism, thereby ensuring complete bioorthogonality and minimizing interference with endogenous biological processes [23].

In this study, we employed a porcine HLI model based on triple ligation—right external iliac artery (EIA) and bilateral internal iliac arteries (IIAs)—an approach that creates extensive ischemia and has been widely used in preclinical studies [5]. This model yields sustained, quantifiable deficits in limb muscle perfusion for at least six weeks, providing a chronic ischemic state with functional impairment at a human-relevant scale. Taken together, these features mirror key aspects of hindlimb ischemia in patients and support the use of this porcine model to evaluate the therapeutic efficacy of our NO delivery system [24].

This study aims to provide insights into the clinical application of exogenous NO as a potential treatment for PAD and to assess the translational potential of the developed endocellulase-based NO delivery system.

## 2. Materials and Methods

### 2.1. Materials

Wild-type C57BL/6J mice, 10-week-old males weighing 20–25 g, were obtained from Beijing SiPeiFu Biotechnology Co., Ltd. (Beijing, China), and Bama miniature pigs, males weighing 20–25 kg, were sourced from Tianjin Bainong Experimental Animal Breeding Technology Co., Ltd., Tianjin, China.

The use of experimental animals was approved by the Animal Experiments Ethical Committee of Nankai University and conducted according to the Guide for Care and Use of Laboratory Animals.

### 2.2. Expression and Purification of Endocellulases

The endocellulase Cel5A-h38 was expressed and purified as reported previously [23,25]. In brief, recombinant *E. coli* BL21 (DE3) strains were utilized to express the target protein. The strains were inoculated into 200 mL of LB medium supplemented with 50 μg/mL kanamycin. Protein expression was induced by adding 1 mM isopropyl-β-d-thiogalactopyranoside (IPTG) when the OD_600_ reached 0.6–0.8, and the culture was incubated at 25 °C for 8 h. After centrifugation at 10,000 rpm for 10 min at 4 °C, the cell pellets were resuspended in 30 mL of sodium phosphate buffer (PBS, pH 7.4). Following sonication and centrifugation, the supernatant was passed through a Ni^2+^-NTA agarose affinity chromatography column (Qiagen, Hilden, Germany). Proteins were eluted with a buffer containing 250 mM imidazole, 0.5 M NaCl, and 20 mM Tris-HCl (pH 8.0). The purified enzymes were concentrated by microfiltration (MW cutoff of 30 kDa, Amicon Ultra, Millipore, Darmstadt, Germany) and exchanged with PBS (pH 7.4) three times. The enzyme concentration was quantified with a BCA protein assay kit (Pierce Biotechnology, Rockford, IL, USA) using bovine serum albumin as the standard.

### 2.3. Enzymatic Assays

To determine the relative activities of wild-type galactosidase (β-gal), A4-β-Gal^H363A^ (H363A) and Cel5A-h38, the fluorescent substrate Gal-MU was used. The enzyme activity in PBS (pH 7.4) was assayed in a total volume of 100 μL containing 10 μM fluorogenic substrate and 0.005 mg/mL β-gal, H363A, or Cel5A-h38, respectively. The mixture was incubated at 37 °C for 30 min, and the fluorescence intensity was recorded with a fluorescence microplate reader (excitation wavelength 360 nm, emission wavelength 460 nm).

### 2.4. Synthesis of β-D-Cellobiosyl–Pyrrolidinyl Diazeniumdiolate Prodrug (Cel2-NO)

Cel2-NO was synthesized according to a previously reported procedure [23]. Briefly, pyridine (2 mL) and Ac2O (2 mL) were added to a solution of cellobiose (1 g, 2.9 mmol) in dry DMF (5 mL). The mixture was stirred at room temperature overnight. The solvent was subsequently removed in vacuo, and the crude residue was purified via chromatography to obtain the acetylated cellobiose.

33% HBr in acetic acid (5 mL) was added dropwise to acetylated cellobiose (1 g, 1.5 mmol) to afford a yellow solution. After the mixture was stirred for 2 h at room temperature, the solvent was removed in vacuo. The crude product was purified by chromatography to obtain the desired compound, peracetylated cellobiosyl bromide. This labile intermediate was directly used in the next step.

Peracetylated cellobiosyl bromide (0.7 g, 1.0 mmol) and PYRRO/NO (0.2 g, 1.3 mmol) were dissolved in dry DMF (10 mL) under an argon atmosphere. The mixture was stirred for 24 h at room temperature. After the reaction was complete, the mixture was extracted with EtOAc. The combined organic phase was dried over Na_2_SO_4_ and removed under reduced pressure. The residue was purified by chromatography to give the desired compound as a solid. The compound was subsequently dissolved in dry methanol (10 mL), and a catalytic amount of MeONa was added. After the reaction was complete, the solvent was removed under reduced pressure. The obtained crude product was purified by chromatography to give the desired compound Cel2-NO as a white solid (Figure S1).

### 2.5. Preparation of the Cel5A-h38-Loaded HA Hydrogel

Cel5A-h38-loaded HA hydrogel was fabricated via a previously reported method [26]. In brief, HA-CD (30 mg) was added to 1 mL of sterile PBS and allowed to stand overnight for swelling and dissolution. On the following day, 3 mg of HA-AD was added, and the mixture was allowed to sit for 2 h to form the gel. Upon mixing, they rapidly and spontaneously assembled into a hydrogel, which was then centrifuged to remove air and transferred to a syringe. The final concentration of Cel5A-h38 in the mixed solution was 2.0 mg/mL. The HA hydrogel without the enzyme was prepared via the same procedure but with an equivalent volume of PBS.

### 2.6. Characterization of the Cel5A-h38-Loaded HA Hydrogel

The structure and morphology of the hydrogel were observed by scanning electron microscopy (SEM, HITACHI, X-650, Tokyo, Japan). The prepared hydrogel was placed in liquid nitrogen for rapid freezing. Then, the frozen hydrogel was transferred to a freeze dryer for vacuum freeze-drying. After freeze-drying, the hydrogel was cut into small pieces, attached to the sample holder with conductive adhesive, gold-coated, and observed by SEM.

The rheological properties of the prepared hydrogel were studied via a 25 mm parallel plate rheometer (TA Instruments, New Castle, DE, USA). The hydrogel underwent repeated cycles of low (1%) and high (100%) strains at a frequency of 6.0 rad/s. The parameters were adjusted for frequency scanning with a fixed strain of 1%, and the angular frequency was set to range from 0.1 to 100 rad/s.

### 2.7. Detection of Nitric Oxide Release In Vitro

*In vitro* NO release was first determined via a Griess kit assay. 50 µM of NO prodrugs (Gal-NO and Cel2-NO) were dissolved in PBS (pH 7.4), and enzymes (β-gal, H363A, and Cel5A-h38) were added to the solutions at a final concentration of 5 µg/mL. At each time interval, 50 μL of mixture was transferred into a 96-well plate, and then 50 μL of Griess I and Griess II reagents were added thereafter. Finally, the absorbance was measured at a wavelength of 540 nm via a microplate reader.

The real-time release of NO from Cel2-NO was measured via a chemiluminescence NO analyzer (NOA) (Seivers 280i, Boulder, CO, USA). Briefly, 10 mL of PBS solution containing 50 µM Cel2-NO was initially added to the reaction chamber. Following a 2 min baseline calibration of NO levels, Cel5A-h38 (50 µg/mL) was injected into the reaction chamber, and the NO signal was continuously recorded over time.

### 2.8. Immunoassay of Cel5A-h38

The inflammatory response of Cel5A-h38 was evaluated through two independent immunoassays in mice, adequately addressing the goal of preliminary safety assessment. C57BL/6J mice were randomly divided into four groups: the Sham, β-gal, H363A and Cel5A-h38 groups. n = 4 for the sham group and n = 8 for other groups. The mice in each group were intravenously injected with 100 U/kg saline, β-gal, H363A, or Cel5A-h38 via the tail vein. Injections were administered every two days for a total of 7 days. On day 7, blood was collected using heparin as an anticoagulant. The blood was centrifuged at 3000 rpm for 15 min, and the supernatant was collected as plasma, which was then stored at −20 °C. The plasma levels of IL-6 and TNF-α were quantitatively measured via commercially available ELISA kits (Mlbio, Shanghai, China).

In a separate experiment, C57BL/6J mice were randomly divided into two groups: the control and Cel5A-h38 groups, with 6 mice in each group. The mice in each group were intramuscularly administered Cel5A-h38 (5 mg/kg) or PBS. Serum was collected weekly on specific days (day 0, 1, 3, 5, 7, 10, 14, 21 and 28) for complement C3a detection. The serum levels of complement C3a were quantitatively measured using ELISA kits (Njjcbio, Nanjing, China).

### 2.9. NIR Fluorescence Imaging

*In vivo* NIR fluorescence imaging was used to evaluate the specificity of Cel5A-h38 for the endoglucanase-activatable NIR fluorescence imaging probe (EANP). C57BL/6J mice (n = 3) were subcutaneously injected with hydrogel containing Cel5A-h38 (500 μg/mL, 100 μL). Prior to the administration of EANP, fluorescence images were captured with a 1 s acquisition time (excitation wavelength: 675 nm; emission wavelength: 720 nm). Subsequently, EANP (0.25 mM, 100 μL) was intravenously administered 5 min before imaging. Finally, fluorescence images were acquired again under identical imaging conditions. The fluorescence intensity in each image was quantitatively analyzed by defining regions of interest (ROIs) via Living Image software (version 4.5.2, PerkinElmer, Waltham, MA, USA).

An NO fluorescence probe (DAC-S) was used to detect the production of NO in the hindlimbs of the mice [27]. C57BL/6J mice (n = 3) were subcutaneously injected with a hydrogel containing Cel5A-h38 and DAC-S. Prior to the administration of the NO prodrug, fluorescence images were captured with a 1 s acquisition time (excitation wavelength: 640 nm; emission filter: Cy5). The NO prodrug (1 mg/mL, 100 μL) was subsequently intravenously administered 5 min before imaging. Finally, fluorescence images were acquired again under identical imaging conditions. The fluorescence intensity in each image was quantitatively analyzed by defining the ROIs via Living Image software.

### 2.10. Detection of Nitric Oxide Release In Vivo

The electronic paramagnetic resonance (EPR) assay was used to measure NO levels in the muscles of the lower extremities. In brief, 100 μL of Cel5A-h38-loaded HA hydrogel (500 μg/mL) was implanted into the hindlimb of the mice one day prior. On the second day, the mice were anesthetized with isoflurane, followed by an intraperitoneal injection of DETC sodium salt (500 mg/kg, Sigma—Aldrich, Saint Louis, MO, USA) dissolved in distilled deionized water (250 mM). After 5 min, a subcutaneous injection of ammonium ferric sulfate (50 mM) in citric acid solution (250 mM) (2 mL/kg) was administered. Five minutes later, 100 μL of NO prodrug (1 mg/mL) was injected through the tail vein. One hour later, the hindlimb muscles and tissues from other organs (heart, brain, liver, spleen, lung, and kidney) were collected and frozen in liquid nitrogen. The frozen tissue was then homogenized and immediately extracted with ethyl acetate. The ethyl acetate extract was transferred into a 50 μL capillary tube, and X-band EPR measurements were performed at room temperature (23 ± 1 °C). The EPR instrument settings were as follows: modulation frequency 100 kHz, microwave power 10 mW, modulation amplitude 2 G, and 15 scan repetitions. A standard curve was generated via TEMPO, and the peak height in the EPR spectrum was used to quantify the concentration of NO-Fe (DETC)_2_.

### 2.11. Systemic Cardiovascular Effects of the Cel5A-h38-Cel2-NO Pair

C57BL/6J mice were randomly divided into three groups (n = 3 per group): saline, Gal-NO, and Cel2-NO. Each mouse received a hindlimb injection of Cel5A-h38-loaded hydrogel. The animals were then administered via the tail vein 100 μL of saline, Gal-NO, or Cel2-NO (1 mg/mL) every two days for a total of 7 days. Blood pressure was monitored daily before and after the initiation of treatment. Cardiac and aortic ultrasound imaging were performed at pre-determined timepoints. After 7 days, heart and aortic tissues were harvested for H&E staining.

### 2.12. Tissue Compatibility of the Cel2-NO

C57BL/6J mice were randomly divided into two groups: the control and Cel2-NO groups, with 3 mice in each group. The mice in each group were intravenously injected with 100 μL Cel2-NO (1 mg/mL) or saline via the tail vein. Injections were administered every two days for a total of 7 days. On day 7, blood was collected for blood biochemistry and blood routine analysis. In addition, the hearts, livers, spleens, lungs, and kidneys were excised, processed for histopathological examination, and stained with H&E.

### 2.13. Dose of NO Prodrug in Micropigs

The dosage of the Cel2-NO prodrug was converted from mice to micropigs via the body surface area (BSA) normalization method, which is a standard practice in translational pharmacology and toxicology. This approach employs species-specific Km factors (3 for mice and 27 for micropigs) [28]. The conversion was performed according to the formula: Dose–micropig (mg/kg) = Dose–mouse (mg/kg) × (Km–mouse/Km–micropig). On the basis of a previously established effective mouse dose of 5 mg/kg [23], the equivalent dose for a 20 kg micropig was calculated, resulting in a prodrug formulation of 11 mg/mL in a 1 mL injection volume. The dosage of the Cel5A-h38 enzyme was selected according to our previous studies in order to maintain the local catalytic activity.

### 2.14. Pig Hindlimb Ischemia Model

Bama miniature pigs (20–25 kg) were fasted for 24 h before surgery. Anesthesia was induced with intramuscular midazolam (20 mg/30 kg) and Xylazine hydrochloride (100 mg/30 kg) and maintained with 2% isoflurane via a ventilator. The surgical instruments were sterilized, and the heart rate, oxygen saturation, and temperature were monitored. Blood pressure was recorded on the limbs. The neck and right lower limb were shaved, and local disinfection with iodine was performed. A midline abdominal incision was used to expose the retroperitoneal arteries, and EIA and bilateral IIAs were ligated and excised [24]. The hydrogel with Cel5A-h38 (1 mL, 1 mg/mL) was injected into the muscles around the ligated arteries. The wound was sutured, and local disinfection was performed. A 10 cm neck incision was made to isolate the external jugular vein, and a catheter was placed. NO prodrug (11 mg/mL, 1 mL) was injected every two days via the catheter until day 28. To prevent infection, ceftriaxone sodium (60 mg/kg) was administered. The pigs were monitored until they regained consciousness.

Blood pressure in the front limbs and ischemic hindlimbs of the Bama pigs was monitored via a veterinary blood pressure monitor pre-surgery and at 28 days post-surgery. The ankle–brachial index (ABI) is calculated as hindlimb pressure divided by ipsilateral forelimb pressure.

### 2.15. Imaging Analysis

In this study, Bama pigs underwent imaging analysis via both digital subtraction angiography (DSA) and computed tomography angiography (CTA). Prior to both procedures, the pigs were fasted for more than 24 h and anesthetized via intramuscular injection.

For DSA (Philips, Amsterdam, the Netherlands), after fasting and anesthesia, an aortic puncture was performed with ultrasound guidance to insert a catheter, followed by contrast agent injection for vascular imaging. This procedure was performed post-surgery and 7 days later. Ultrasound guidance ensures accurate catheter placement, minimizing vascular damage. After successful catheter insertion, a contrast agent was injected for vascular imaging. The contrast agent used was iodixanol (320 mgI/mL), with a flow rate of 3 mL/s, a total volume of 20 mL, and a pressure of 300 PSI.

CTA was performed at 28 days post-surgery. For CTA (GE Healthcare, Waukesha, WI, USA), after fasting and anesthesia, CT scans were performed with the following parameters: tube voltage of 120 kV, slice thickness of 5 mm, reconstruction thickness of 0.625 mm, detector collimation of 80 mm, pitch of 0.992, spiral scan thickness of 5 mm, and rotation speed of 0.5. Real-time automatic tube current modulation was used, scanning from the pig’s skull to the aortic arch. Contrast agent (iodixanol, 350 mg/dL iodine) was injected intravenously through the ear vein with a high-pressure injector at 1 mL/kg and 2.5 mL/sec for 30–42 mL. 40 mL saline was injected post-contrast at the same rate, and CTA images were then obtained for analysis.

### 2.16. Histological Analysis

Four weeks post-surgery, pigs were anesthetized with isoflurane, and hindlimb muscles were harvested for histological analysis. For frozen sections, samples were dehydrated in 30% sucrose overnight, embedded in optimal cutting temperature (OCT) compound, and cut into 5 μm thick cryosections. Paraffin sections were prepared by fixing in 4% paraformaldehyde overnight, dehydrating in ethanol, clearing with xylene, embedding in paraffin, and sectioning into 5 μm slices. After deparaffinization, H&E staining (Leagene, Beijing, China, DH0020), Masson’s Trichrome staining (Leagene, DC0034), Sirius Red staining (Leagene, DC0041), and fibronectin immunohistochemistry (1:100, ab2413, Abcam, Cambridge, UK) were performed. Images were captured at 100× magnification via an upright microscope, and quantitative analysis was performed via ImageJ 1.48v software (NIH) (≥6 images per animal).

For immunofluorescence, frozen sections were fixed in ice-cold acetone, permeabilized with 0.1% Triton, blocked with 5% normal goat serum, and incubated overnight at 4 °C with primary antibodies (rabbit anti-iNOS, ab178945; rabbit anti-CD206, ab64693; mouse anti-CD31, ab64543; and mouse anti-α-SMA, ab7817). Secondary antibodies (FITC- or rhodamine-conjugated goat anti-mouse IgG or goat anti-rabbit IgG, Invitrogen) were used. Images were captured at 200× magnification with a fluorescence microscope (Zeiss Axio Imager Z1, Jena, Germany), and quantitative analysis was performed with Adobe Photoshop (version CC 2019, Adobe) (≥6 images per animal).

To assess reactive oxygen species (ROS) generation in ischemic limb muscles, dihydroethidium (DHE) staining was performed on frozen sections. Briefly, sections were washed twice in PBS for 5 min each to remove impurities. Then, sections were incubated with 20 μM DHE solution at 37 °C for 30 min in the dark. Following incubation, unbound dye was removed by washing the sections six times in PBS (5 min per wash). Sections were then mounted with DAPI and visualized using an upright fluorescence microscope. Quantitative analysis was conducted using ImageJ.

### 2.17. RNA Extraction and RT—qPCR

At 28 days post-surgery, the pigs were sacrificed, and the gastrocnemius muscles were collected. A 20 mg tissue sample was weighed, and 1 mL of TRIzol reagent (Invitrogen, Carlsbad, CA, USA) was added for total RNA extraction. The RNA concentration was measured via a NanoDrop 2000 spectrophotometer (ThermoFisher Scientific, Waltham, MA, USA). Then, 1 μg of total RNA was reverse transcribed into cDNA via a reverse transcription kit. Quantitative real-time PCR (qPCR) was conducted via the SYBR green method to measure the mRNA expression of target genes, including interleukin 6 (IL6), tumor necrosis factor (TNF), arginase 1 (ARG1), interleukin 4 (IL4), nitric oxide synthase 3 (NOS3), platelet endothelial cell adhesion molecule 1 (PECAM1) and vascular endothelial growth factor A (VEGFA). The 2−(ΔΔCT) method was used to calculate the relative expression levels of the target mRNAs, which were then normalized to the expression of GAPDH. The primer sequences are provided in Table 1.

### 2.18. Western Blot Analysis

Pig hindlimb muscles were harvested in RIPA lysis buffer. The protein concentration was quantified by using a BCA Kit (Solarbio, Beijing, China). Proteins were subsequently separated via 10% SDS—PAGE and transferred onto PVDF membranes (Millipore). The membranes were blocked in 5% non-fat milk for 1 h at room temperature. The samples were incubated with primary antibodies overnight at 4 °C. Mouse anti-CD31 (Abcam, ab64543, 1:1000), rabbit anti-eNOS (Invitrogen, PA1-037, 1:1000), rabbit anti-VEGFA (Wanlei, Shenyang, China, WL00009b, 1:1000), rabbit anti-IL-10 (ABclonal, Wuhan, China, A12255, 1:1000), rabbit anti-IL-6 (ABclonal, A11114, 1:1000), rabbit anti-IL-4 (ABclonal, A24733, 1:1000), rabbit anti-iNOS (Abcam, ab178945, 1:1000), rabbit anti-CD206 (Abcam, ab64693, 1:1000) and rabbit anti-TNF-α (ABclonal, A22227, 1:1000) rabbit anti-fibronectin (Abcam, ab2413, 1:1000), rabbit anti-TGF-β1 (ABclonal, A23262, 1:1000), and rabbit anti-P-Smad2/3 (ABclonal, AP0548, 1:1000) antibodies were used in this work. Horseradish peroxidase (HRP)-conjugated secondary antibodies (ZSGB-Bio, Beijing, China, ZB-2301, 1:2000) were used as secondary antibodies. Signals were visualized with an enhanced chemiluminescence (ECL) substrate. GAPDH (Proteintech, Wuhan, China, 60004-1-Ig, 1:1000) was used as an internal control.

### 2.19. Statistical Analysis

All the data, representing the mean ± SEM from a minimum of three independent experiments, were analyzed via two-tailed Student’s *t* tests for two groups. Comparisons among more than two groups were made by one-way or two-way analysis of variance (ANOVA) followed by Tukey’s post hoc analysis for multiple pairwise comparisons. Prism 10.0 GraphPad software (San Diego, CA, USA) was used for these analyses. Differences were considered to be significant at *p* <  0.05.

## 3. Results and Discussion

### 3.1. Construction and Characterization of a Precise Delivery System for Nitric Oxide Using Endocellulase

**Figure 1:**
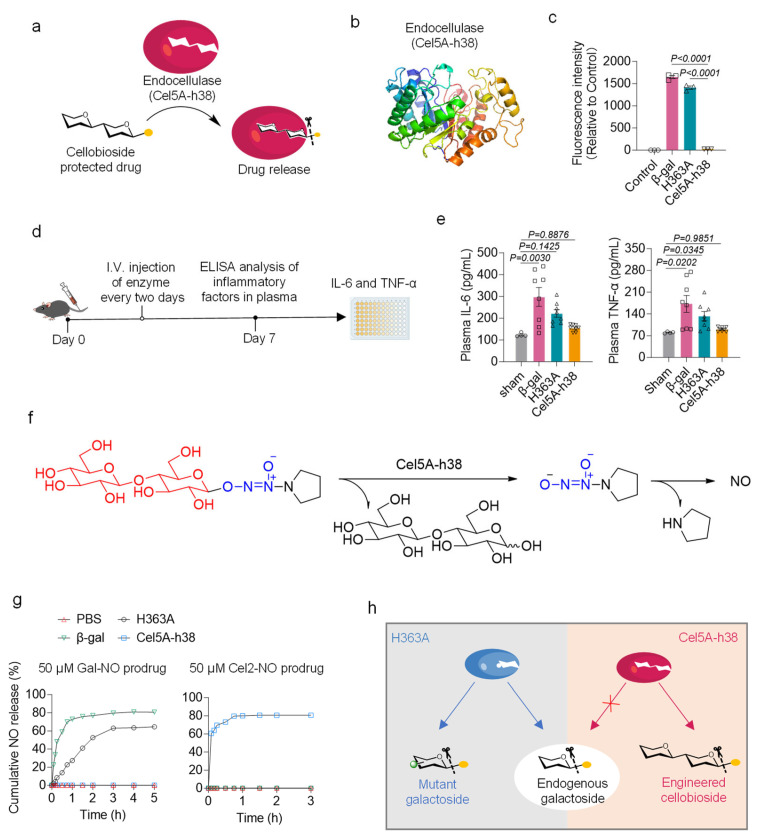
Construction and characterization of an enzyme–prodrug pair based on the endocellulase for the controlled delivery of nitric oxide (NO). (**a**) Schematic illustration showing the conversion of cellobiose-protected NO prodrugs into NO under the catalysis of endocellulase. (**b**) The ribbon structure of Cel5A-h38 predicted by AlphaFold. (**c**) The catalytic abilities of β-gal, H363A, and Cel5A-h38 on β-galactosidic bonds. Control: fluorescence probe with PBS, β-gal: fluorescence probe with wild-type β-galactosidase; H363A: fluorescence probe with engineered β-galactosidase (A4-β-GalH363A); Cel5A-h38: fluorescence probe with endocellulase (n = 3 per group). (**d**) Schematic representation of the experimental design. (**e**) Plasma IL-6 and TNF-α levels in mice treated with β-gal, H363A, or Cel5A-h38 (n = 4 for the sham group and n = 8 for other groups). (**f**) The mechanism of NO release via the decomposition of Cel2-NO catalyzed by the Cel5A-h38. (**g**) NO generation from Gal-NO and Cel2-NO in response to β-gal, H363A, and Cel5A-h38, respectively (n = 3 per group). (**h**) Schematic illustration depicting the difference between Cel5A-h38-based and H363A-based enzyme–prodrug systems. One-way ANOVA with Tukey’s post hoc analysis was performed. All data are presented as the means ± SEM. Differences were considered to be significant at *p*  <  0.05.

To achieve precise delivery of NO, an enzyme—prodrug pair was constructed using an endocellulase enzyme (Cel5A-h38) and the corresponding NO prodrug (Figure 1a,b). Cel5A-h38 is not involved in human cellular metabolism but is present in bacterial and fungal organisms. It enables complete bioorthogonality and minimizes interference with endogenous biological processes, which is an important advantage over our previously developed enzyme—prodrug system based on galactosidase [20,29]. As shown in Figure 1c, either β-gal or its mutant (H363A) interacts with the galactoside-protected fluorescence probe, resulting in evidently enhanced fluorescence signals. In contrast, Cel5A-h38 demonstrated no enzymatic activity toward the galactoside substrate.

The inflammatory response induced by Cel5A-h38 delivery was initially evaluated in a mouse model. Cel5A-h38 solution was administered to mice via intravenous injection. β-gal and H363A were also administered for comparison. The administration of β-gal or H363A increased the plasma concentrations of inflammatory mediators, as evidenced by increased levels of interleukin-6 (IL-6) and tumor necrosis factor-alpha (TNF-α). These findings are consistent with those of a previous study, indicating that elevated levels of galactosidase may influence IgG galactosylation and potentially induce an inflammatory response [30]. Notably, the levels of inflammatory factors (IL-6 and TNF-α) in the Cel5A-h38 group did not significantly increase (Figure 1d,e). Further assessment of anti-Cel5A-h38 IgG/IgM antibodies [23] and complement C3a confirmed a low immunogenicity profile, with no significant immune response or complement activation detected *in vivo* (Figure S2). To evaluate the catalytic activity of Cel5A-h38 for the conversion of the NO prodrug, NO release was assessed using a Griess assay. These results indicate that both β-gal and H363A were capable of hydrolyzing galactoside-protected NO donors to release NO, whereas Cel5A-h38 lacked catalytic activity. In contrast, when cellobiose-protected NO donors (Cel2-NO) were employed, only Cel5A-h38 was able to catalyze the release of NO, a capability not observed in either β-gal or H363A (Figure 1f,g). Overall, the enzyme—prodrug pair consisting of Cel5A-h38 and Cel2-NO represented a fully bioorthogonal delivery system that did not induce side effects *in vivo* (Figure 1h).

### 3.2. Targeted Delivery of Nitric Oxide In Vivo

**Figure 2:**
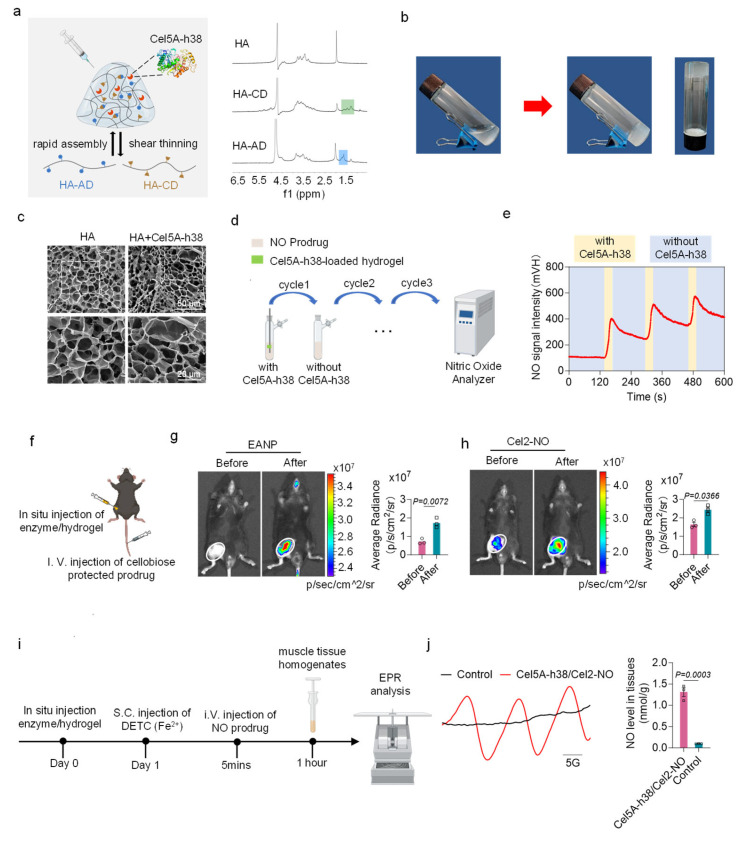
Targeted delivery of nitric oxide *in vivo* by endocellulase-based delivery system. (**a**) Schematic diagram showing the composition of the Cel5A-h38-loaded HA hydrogel. NMR spectra of HA, HA-AD, and HA-CD are shown. (**b**) Optical images showing the transition from HA solution into the hydrogel. (**c**) Representative scanning electron microscopy (SEM) images of HA hydrogel and Cel5A-h38-loaded HA hydrogel. (**d**) Experimental schedule for the measurement of NO release from the Cel2-NO prodrug catalyzed by Cel5A-h38-loaded hydrogel. (**e**) Release of NO from Cel2-NO in the presence and absence of Cel5A-h38-loaded HA hydrogel. (**f**) Schematic illustration of the fluorescence imaging assay for the detection of *in vivo* catalytic property of Cel5A-h38. (**g**) Representative images showing the specific hydrolysis of endoglucanase-activatable NIR fluorescence imaging probe (EANP) in response to Cel5A-h38 in the mouse hindlimb with quantitative analyses of the fluorescence intensity in the target site (n = 3 per group). (**h**) Representative images showing the in situ NO generation from NO prodrug by the Cel5A-h38-loaded HA hydrogel with quantitative analyses of the fluorescence intensity in the target site (n = 3 per group). (**i**) Experimental schedule for the electron paramagnetic resonance (EPR) assay for the detection of NO generation. (**j**) Representative EPR spectra reflecting NO generation within the hindlimb tissue in the presence of (DETC)2Fe (left). The double integrated area of the spectrum was calibrated into concentration using Tempol as a standard (right), n = 3 per group. All data are presented as the means ± SEM. Unpaired two-tailed Student’s *t* test was performed. Differences were considered to be significant at *p*  <  0.05.

For the *in vivo* delivery of NO, an injectable HA hydrogel was utilized as the carrier of Cel5A-h38. The hydrogel consists of two components—adamantane-modified hyaluronic acid (HA-AD) and β-cyclodextrin-modified HA (HA-CD)—that enable in situ gelation via host—guest interactions between CD and AD (Figure 2a,b). SEM images revealed that both the HA hydrogel and the Cel5A-h38-loaded HA hydrogel exhibited an interconnected porous network structure without detectable differences (Figure 2c). The rheological properties of the hydrogel were evaluated by applying low (1%) and high (100%) strain oscillations, and changes in the storage modulus (G′, red symbols) and loss modulus (G″, blue symbols) were observed (Figure S3). The hydrogel behaves as a liquid under high shear stress but rapidly transforms into a gel under low shear stress. The hydrogel employed in this study is based on a natural polymer, HA, with optimal biocompatibility. As an enzyme carrier, the HA hydrogel effectively enhances the local retention and stability of Cel5A-h38 at the ischemic site. The engineered delivery system thus prepared enables sustained NO release, demonstrating good economic feasibility and translational potential.

The catalytic activity of the Cel5A-h38-loaded HA hydrogel was monitored using a chemiluminescence NO analyzer. The prodrug Cel2-NO decomposed rapidly, resulting in the accumulation of the NO signal when it interacted with the hydrogel, whereas the signal decreased gradually upon removal of the hydrogel. It shows good repeatability after three cycles (Figure 2d,e). The release of NO from Cel2-NO under the catalysis of the Cel5A-h38-loaded HA hydrogel was further assessed using a Griess assay (Figure S4). The specificity and catalytic activity of Cel5A-h38 were evaluated by *in vivo* imaging using a cellobiose-protected near-infrared fluorescence probe in a mouse model [23]. The Cel5A-h38-loaded hydrogel was first injected into the hindlimbs of the mice, followed by intravenous administration of the probe. A marked increase in fluorescence intensity was observed specifically at the site of the hydrogel, reflecting the precise and targeted delivery of the prodrug (Figure 2f,g). Similarly, *in vivo* NO generation from the prodrug Cel2-NO was detected using an NO fluorescence probe (DAC-S) [31,32] that was also loaded into the hydrogel (Figure 2h). *In vivo* NO release was further evaluated by EPR using (DETC)_2_Fe^2+^ as a spin trapping reagent [33]. Following the administration of Cel2-NO, the EPR spectra recorded from the hindlimb implanted with Cel5A-h38 exhibited a characteristic triplet signal, corresponding to the (DETC)_2_Fe-NO adduct (Figure 2i,j). Furthermore, NO levels at the targeted site (hindlimb implanted with Cel5A-h38) were significantly higher (*p* < 0.0001) than those in non-target tissues, including the control hindlimb, heart, brain, liver, spleen, lung, and kidney (Figure S5). These results confirm that our enzyme–prodrug system enables spatially controlled NO release with minimal systemic distribution, supporting its localized mode of action. In addition, in contrast to the systemic NO delivery (Gal-NO), the localized NO delivery prevented the fluctuations in systemic blood pressure. Ultrasound and histological analyses confirmed the absence of adverse effects on the functions of the heart and blood vessels (Figure S6). Similarly, in pig models, systemic blood pressure maintained stability after localized NO delivery (Figure S7).

Finally, histological examination and blood analyses revealed no significant differences between mice treated with saline and Cel2-NO (Figure S8), indicating favorable tissue compatibility.

### 3.3. Targeted Delivery of Nitric Oxide Ameliorates Hindlimb Ischemia in a Pig Model

**Figure 3:**
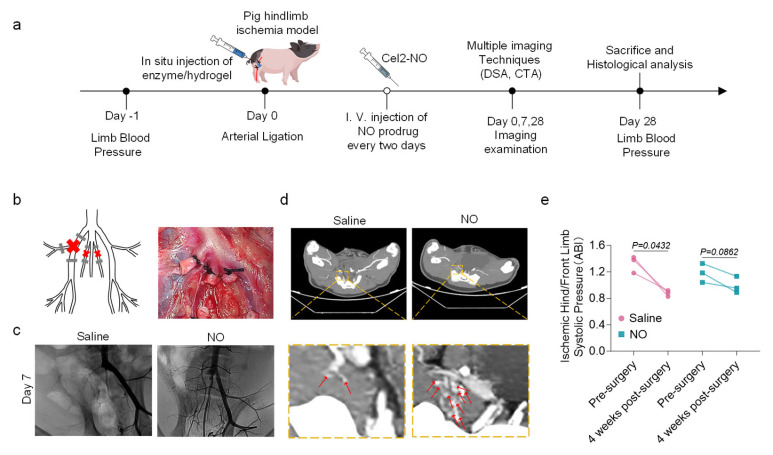
Targeted delivery of nitric oxide ameliorates tissue ischemia in a pig model. (**a**) Experimental schedule for the evaluation of therapeutic efficacy of NO in a pig model. (**b**) Schematic illustrations of the pig hindlimb ischemia model with representative images of the ligated sites. (**c**) Representative images of Digital Subtraction Angiography (DSA) at 7 days. (**d**) Representative images of Computed Tomography Angiography (CTA) at 28 days. Red arrows indicate sprouting angiogenesis. (**e**) The ankle–brachial blood pressure indexes for ischemic hindlimb before and 4 weeks after the surgery (n = 3 per group). All data are presented as the means ± SEM. Paired two-tailed Student’s *t* test was performed. Differences were considered to be significant at *p*  <  0.05.

The therapeutic efficacy of the Cel5A-h38-based NO delivery system was systematically evaluated in a preclinical porcine model of hindlimb ischemia [34,35] (Figure 3a). The model was established by ligating and severing the right EIA and bilateral IIAs [24], a procedure described as a robust HLI paradigm and effective in mimicking common clinical arterial occlusion and the resulting restricted blood flow. DSA confirmed blood flow obstruction, with no vessels detectable in the ischemic region, indicating blood supply insufficiency (Figure 3b and Figure S9).

During the early postoperative phase, detection was aimed at collateral formation and neovascularization, a period characterized by irregular vessel walls and unstable blood flow in small vessels. DSA was selected for its high resolution and sensitivity in visualizing dynamic blood flow, making it particularly suitable for identifying nascent vessels at this stage [36]. At 7 days post-surgery, DSA images revealed increased blood flow and the formation of new collateral vessels around the ligation site in the NO group (Figure 3c).

By the late postoperative period (28 days), the assessment focus was expanded to include not only neovascularization but also the overall vascular architecture and surrounding tissues, for which CTA provided more comprehensive structural information. CTA analyses further demonstrated robust new vessel formation at the site of arterial severance in the NO group. The newly formed vessels were greater in number and more widely distributed in the NO group than in the control group, with minimal vascularization (Figure 3d).

NO levels in plasma and muscle tissues were measured at 28 days post-surgery. The plasma NO levels were not significantly different between the two groups, whereas the NO level in hindlimb muscle tissues was markedly greater in the NO group than in the control group, confirming that the targeted delivery of NO is controlled by endocellulase without changing the systemic NO distribution (Figure S10).

We measured and calculated the ABI, which is the core quantifiable indicator, preoperatively and at 4 weeks post-surgery in accordance with the 2024 ACC/AHA Guidelines [37]. The NO group did not show significant changes in blood pressure at 28 days post-surgery, unlike the control group, which exhibited a noticeable decrease (*p* <  0.05), suggesting that NO treatment may facilitate blood flow improvement (Figure 3e).

### 3.4. Targeted Delivery of NO Promotes Neovascularization

**Figure 4:**
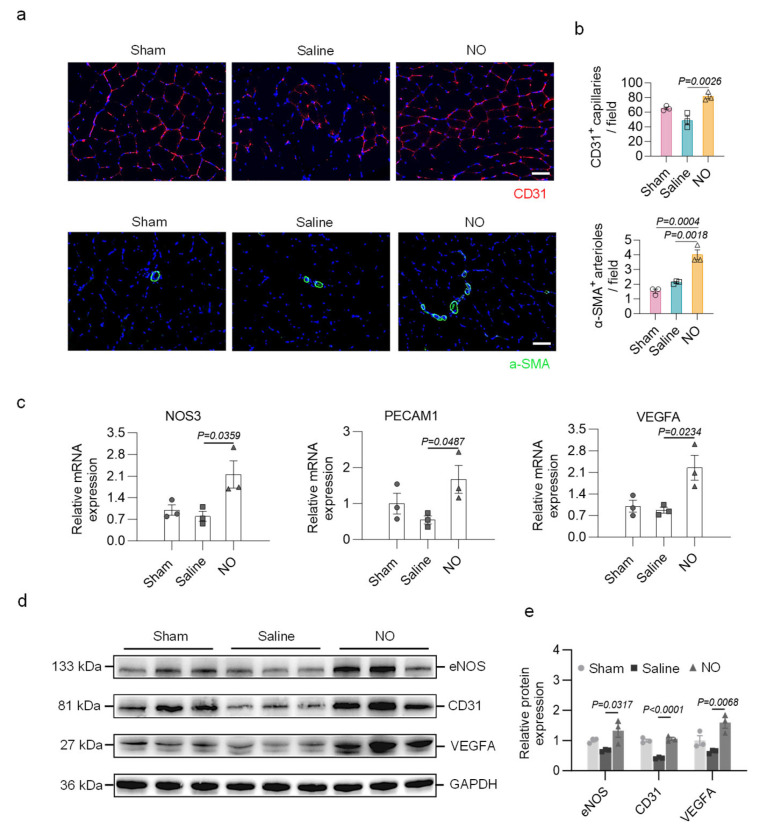
Targeted delivery of NO promotes neovascularization in a pig model of hindlimb ischemia. (**a**) Representative immunofluorescence staining for CD31 (red) and a-SMA (green). Scale bar, 50 μm. (**b**) Quantitative analyses of the density of CD31-positive capillaries and α-SMA-positive arterioles (n = 3 per group). (**c**) The relative expression levels of angiogenesis-related genes (n = 3 per group). (**d**,**e**) Shown is a Western blot and quantification for expression of eNOS, CD31, and VEGFA in the hindlimb muscle from indicated groups (n = 3 per group). One-way ANOVA with Tukey’s post hoc analysis was performed. All data are presented as the means ± SEM. Differences were considered to be significant at *p * <  0.05.

Next, we assessed the impact of targeted delivery of NO on neovascularization, a process essential for tissue growth and functional perfusion recovery. Immunofluorescence staining for CD31, a marker of endothelial cells, revealed that targeted delivery of NO promoted angiogenesis in the damaged area, with higher capillary density observed in the NO group than in the control group at 28 days post-surgery, suggesting that NO promoted angiogenesis and supported blood supply restoration. Targeted delivery of NO also promoted the regeneration of arterioles, as evidenced by the increased number of α-SMA-positive arterioles owing to the pivotal role that NO played in vascular maturation (Figure 4a,b). RT—qPCR and Western blot analyses of muscle tissue further revealed that NO treatment effectively promoted angiogenesis at both the gene and protein levels compared with those in the control group (Figure 4c–e).

In general, the pathology of PAD involves reduced vessel density as well as prevalent dysfunctional microvasculature. While VEGF-based therapy drives angiogenesis, it often produces leaky, immature vessels due to insufficient pericyte recruitment and abnormal vascular morphology [38,39,40,41]. As a multi-functional signaling molecule, NO can promote vascular stabilization by recruiting mural cells and facilitating branched network formation, in which the NO-sGC axis plays a critical role in enhancing endothelial–pericyte interactions and preventing leakage [42,43].

### 3.5. Targeted Delivery of NO Modulates the Inflammatory Response and Suppresses Oxidative Stress

**Figure 5:**
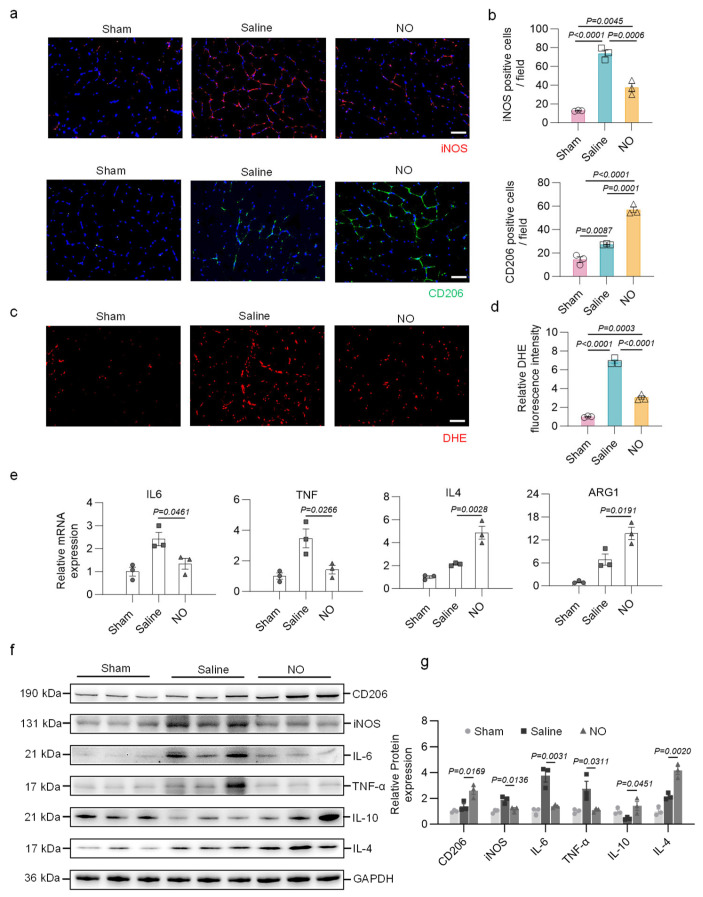
Targeted delivery of NO facilitates the polarization of macrophages towards the M2 phenotype and suppresses the generation of reactive oxygen species (ROS). (**a**) Representative immunofluorescence staining for iNOS (red) and CD206 (green). Scale bar, 50 μm. (**b**) Quantitative analyses of CD206-positive cells and iNOS-positive cells (n = 3 per group). (**c**,**d**) Representative images of DHE staining and quantification of ROS level in the hindlimb muscle (n= 3 per group); scale bar, 50 μm. (**e**) The relative expression levels of inflammation-related genes (n = 3 per group). (**f**,**g**) Shown is a Western blot and quantification for expression of CD206, iNOS, IL-6, TNF-α, IL-10 and IL-4 in the hindlimb muscle from indicated groups (n = 3 per group). One-way ANOVA with Tukey’s post hoc analysis was performed. All data are presented as the means ± SEM. Differences were considered to be significant at *p*  <  0.05.

Inflammation is deeply involved in the formation and progression of PAD [44,45]. The results of the immunofluorescence staining demonstrated that targeted NO release reduced the infiltration of iNOS^+^ cells (M1 macrophages) while increasing the infiltration of CD206^+^ cells (M2 macrophages) in ischemic areas, which is consistent with previous studies showing that NO can regulate inflammation by promoting the polarization of macrophages toward an anti-inflammatory and pro-regenerative M2 phenotype instead of a proinflammatory M1 phenotype [46,47,48] (Figure 5a,b). DHE staining further revealed that NO-treated muscle exhibited lower ROS content compared to the control group (Figure 5c,d). RT—qPCR analyses of gastrocnemius muscle tissue revealed that NO treatment downregulated the expression of the proinflammatory genes IL6 and TNF while increasing the expression of the anti-inflammatory genes IL4 and ARG1 (Figure 5e). These findings were further supported by the Western blot results; that is, the levels of proinflammatory proteins, including IL-6, TNF-α, and iNOS, were decreased, whereas those of anti-inflammatory proteins, including IL-4, IL-10, and CD206, were increased in the NO group (Figure 5f,g). Overall, the targeted delivery of NO can suppress local inflammatory responses and oxidative stress after ischemic injury, which is favorable for subsequent tissue repair and functional recovery.

### 3.6. Targeted Delivery of NO Inhibits Muscle Fibrosis

**Figure 6:**
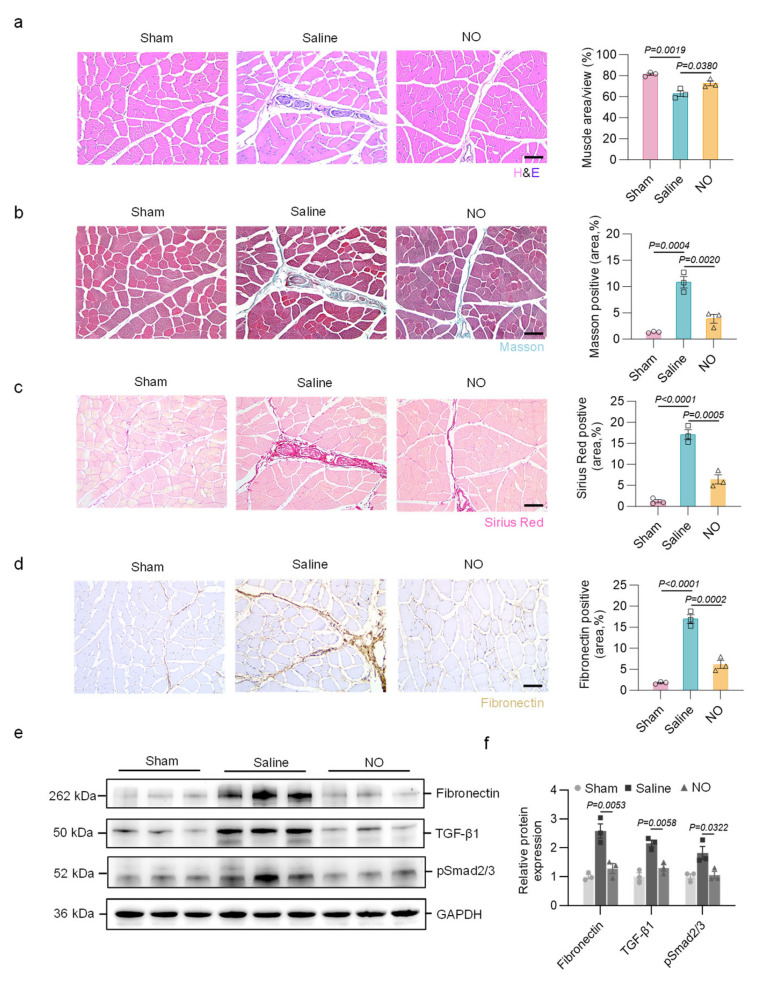
Targeted delivery of NO inhibits muscle fibrosis in a pig model of hindlimb ischemia. (**a**) Representative images of H&E staining and quantification of muscle area in hindlimb muscle (n = 3 per group). Scale bar, 100 μm. (**b**) Representative images of Masson staining and quantification of positive area (n = 3 per group). Scale bar, 100 μm. (**c**) Representative images of Sirius Red staining and quantification of positive area (n = 3 per group). Scale bar, 100 μm. (**d**) Representative immunohistochemical staining for fibronectin and quantification of fibronectin-positive area (n = 3 per group). Scale bar, 100 μm. (**e**,**f**) Shown is a Western blot and quantification for expression of fibronectin, TGF-β1, and pSmad2/3 in the hindlimb muscle (n = 3 per group). One-way ANOVA with Tukey’s post hoc analysis was performed. All data are presented as the means ± SEM. Differences were considered to be significant at *p*  <  0.05.

H&E staining was performed to evaluate muscle damage and fibrosis. At 28 days post-surgery, muscle cells showed remarkable degeneration with inflammatory cell infiltration due to ischemic injury caused by artery ligation. NO treatment efficiently alleviated these changes, indicating the function of NO in regulating tissue repair and inflammatory responses (Figure 6a).

Fibrosis was further assessed by Masson’s trichrome and Sirius Red staining, which revealed that collagen deposition was significantly reduced after NO treatment compared with that in the control group (Figure 6b,c). Immunohistochemistry revealed fibronectin-positive staining in fibrotic areas in the control group, indicating excess extracellular matrix deposition. In contrast, NO treatment inhibited fibrosis progression and matrix deposition, as illustrated by the reduced fibronectin-positive area (Figure 6d). Western blot analyses confirmed that targeted delivery of NO inhibited the expression of fibrosis-associated proteins and interfered with the canonical Smad signaling pathway of TGF-β1 in ischemic tissues (Figure 6e,f).

### 3.7. Study Limitations

This study has several limitations. First, the porcine HLI model was created by acute arterial ligation, which differs from the gradual, multifactorial progression typical of human PAD [24,49,50,51]. Common comorbidities (advanced age, diabetes, hypertension, smoking) were not modeled [52], and overt tissue loss/necrosis—a defining feature of CLTI—was not observed; accordingly, this study should be interpreted as a preclinical large-animal model of HLI with restricted flow rather than a full CLTI model. Second, sample sizes were necessarily modest in the large-animal arm, limiting the power to resolve small-to-moderate effects and inter-individual variability; in addition, the experiments used male minipigs only, precluding analyses of sex-specific responses. Finally, local enzyme deposition was performed by surgical intramuscular injection around ligated arteries; translating this step to humans will require the development of minimally invasive delivery methods (e.g., percutaneous or catheter-guided) and operational workflows compatible with clinical practice.

## 4. Conclusions

In conclusion, the precise delivery of NO was achieved using an enzyme—prodrug delivery system based on endocellulase, and the therapeutic efficacy toward hindlimb ischemia was evaluated in a clinically relevant porcine model. The targeted delivery of NO to lesion sites effectively improved blood perfusion, promoted neovascularization, reduced inflammation and fibrosis, and ultimately facilitated the repair of ischemic muscle. This study not only illustrates the substantial potential of precise NO delivery in the clinical management of PAD but also underscores the translational significance of endocellulase-based delivery systems in clinical applications.

## Figures and Tables

**Table 1:** Primer sequences used for qPCR.

Gene Name	Forward Primer (5′ to 3′)	Reverse Primer (5′ to 3′)
IL6	GCTGCTTCTGGTGATGGCTACTGCC	TGAAACTCCACAAGACCGGTGGTGA
TNF	ATGAGCACTGAGAGCATGATCCG	CCTCGAAGTGCAGTAGGCAGA
IL4	GCGAGAAAGAACTCGTGCATGG	CTCAGGAGGCTCTTCATGCAC
ARG1	AGCCTGTGTCTTTTCTCCTGA	GTCCACGTCTCTCAGGCC
VEGFA	GCCCACTGAGGAGTTCAACATC	GGCCTTGGTGAGGTTTGATC
NOS3	GGAAGCTGCAGGTGTTCGAT	CGGTTGGTGGCGTACTTGAT
PECAM1	CCGAGGTCTGGGAACAAAGG	AGCCTTCCGTTCTAGAATATCTGTT
GAPDH	TTGTGATGGGCGTGAA	TCTGGGTGGCAGTGAT

## Data Availability

The original contributions presented in this study are included in the article/Supplementary Materials. Further inquiries can be directed to the corresponding authors.

## Supplementary Materials

The following supporting information can be downloaded at https://www.mdpi.com/article/10.3390/bioengineering13020128/s1, Figure S1: Synthetic routes of Cel2-NO; Figure S2: Detection of Complement C3a concentration in serum by ELISA following injection of Cel5A-h38 or PBS (n = 6 per group); Figure S3: Rheological properties of HA hydrogel; Figure S4: *In vitro* NO release profile of Cel2-NO triggered by Cel5A-h38-loaded hydrogel measured by Griess assay (n = 3 per group); Figure S5: NO levels in different tissues after administration of Cel5A-h38/Cel2-NO pair in a mouse hindlimb model; Figure S6: Evaluation of the systemic cardiovascular effects of the Cel5A-h38–Cel2-NO pair in a mouse model; Figure S7: Effects of NO treatment on porcine forelimb systolic blood pressure (SBP); Figure S8: Tissue compatibility of the NO prodrug; Figure S9: Representative surgical image (pre-ligation) and post-procedure angiogram (after contrast injection in the distal aorta) for the triple ligation model; Figure S10: The levels of NO in plasma and tissues at 28 days.
