# Supplementary material for: Precise Delivery of Nitric Oxide Controlled by Bioorthogonal Endocellulase Ameliorates Hindlimb Ischemia
Source: Bioengineering (Basel). 2026 Jan 23;13(2):128. doi: 10.3390/bioengineering13020128 (PMC12937777; doi:10.3390/bioengineering13020128)
Supplement: Supplementary file 1 [file bioengineering-13-00128-s001.zip › bioengineering-4088303-supplementary.pdf]

Supplementary Materials

Title: Precise Delivery of Nitric Oxide Controlled by Bioorthogonal Endocellulase Ameliorates Hindlimb Ischemia

Page number

Supplemental figures: Page 2 to Page 12

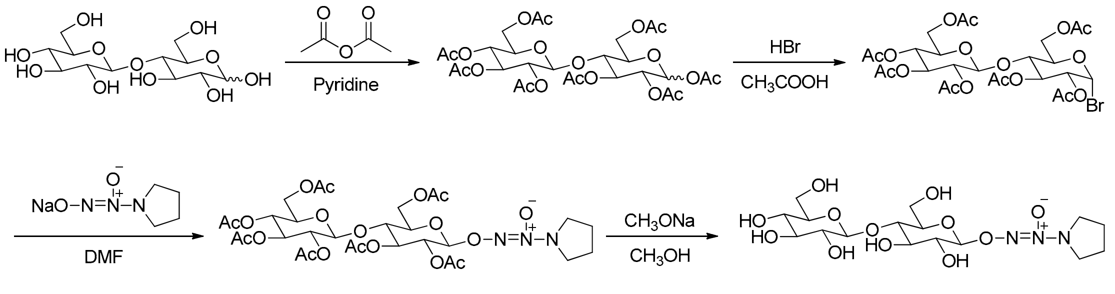

Figure S1. Synthetic routes of Cel2-NO.

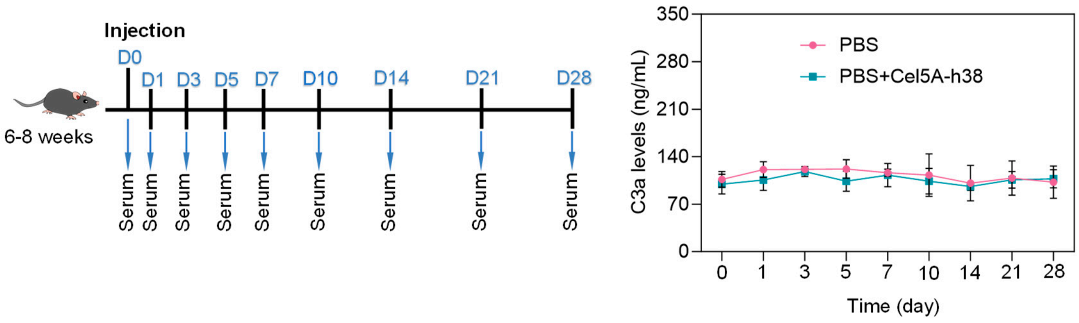

Figure S2. Detection of Complement C3a concentration in serum by ELISA following injection of Cel5A-h38 or PBS (n=6 per group).

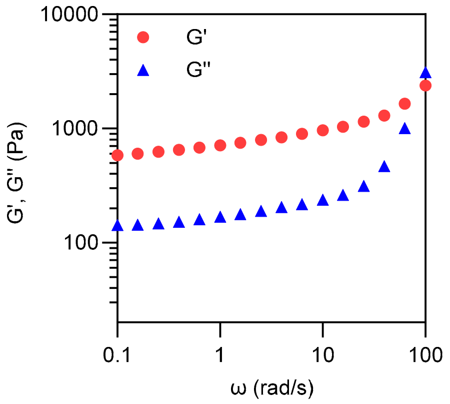

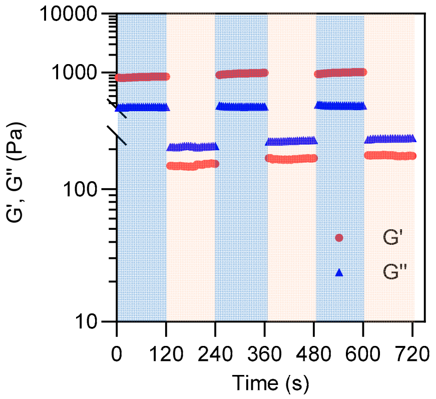

Figure S3. Rheological properties of HA hydrogel. The angular frequency ranges from 0.1 to 100 rad s^{-1}, with a strain of 1%(Left). Repeated cycles of low (1%) strain and high (100%) strain oscillations at 6.0 rad s^{-1}(Right). G′ (red symbols) and G″ (blue symbols) represent storage and loss modulus.

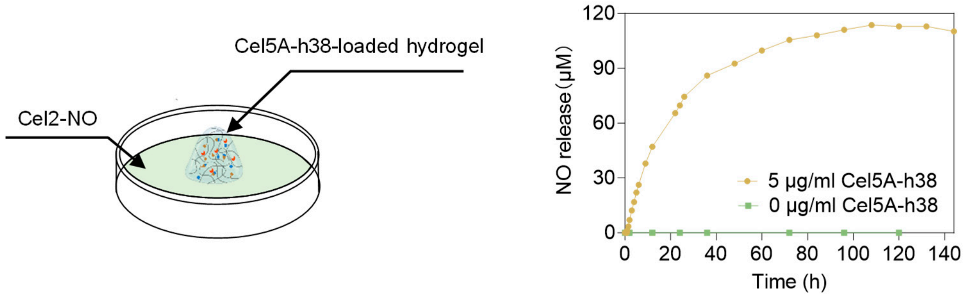

Figure S4. *In vitro* NO release profile of Cel2-NO triggered by Cel5A-h38-loaded hydrogel measured by Griess assay (n =3 per group).

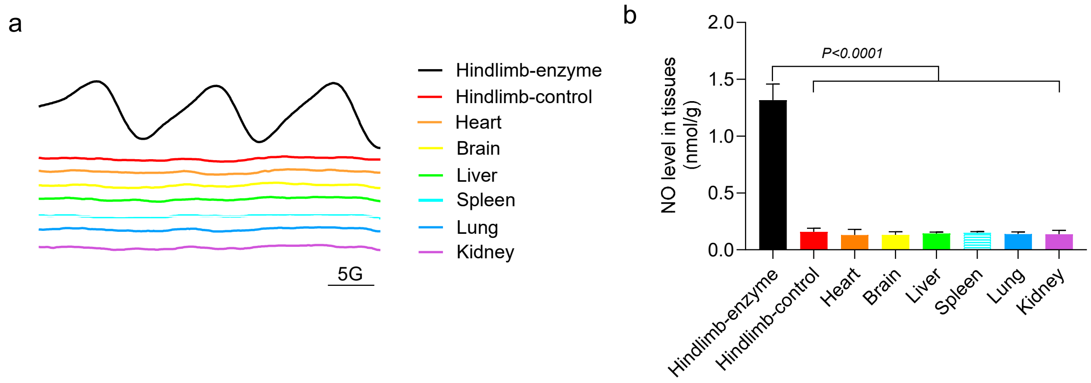

Figure S5. NO levels in different tissues after administration of Cel5A-h38/Cel2-NO pair in a mouse hindlimb model. (a) X-band EPR spectra of organic extracts from various tissues of mice were measured at room temperature. (b) The double integrated area of the spectrum was calibrated into concentration using Tempol as a standard. n=3 per group. One-way ANOVA with Tukey's post-hoc analysis was performed. All data are presented as the means ± SEM. $p < 0.0001$ vs Hindlimb-enzyme.

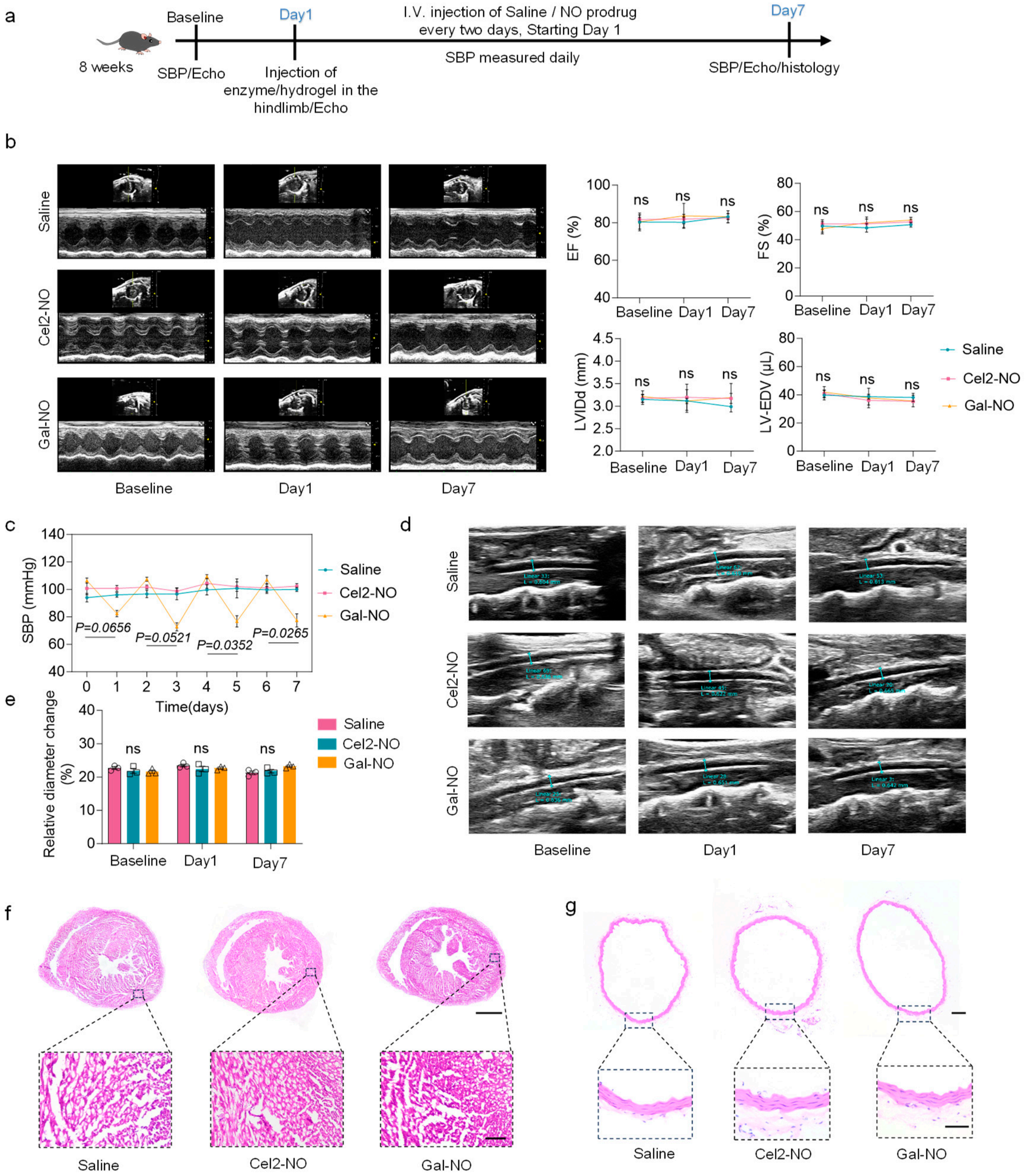
a
8 weeks
Baseline
SBP/Echo
Day1
Injection of enzyme/hydrogel in the hindlimb/Echo
I.V. injection of Saline / NO prodrug every two days, Starting Day 1
SBP measured daily
Day7
SBP/Echo/histology
b
Saline
Cel2-NO
Gal-NO
Baseline
Day1
Day7
EF (%)
FS (%)
LVIDd (mm)
LV-EDV (μL)
ns
c
SBP (mmHg)
Time(days)
P=0.0656
P=0.0521
P=0.0352
P=0.0265
d
e
Relative diameter change (%)
f
g

Figure S6. Evaluation of the systemic cardiovascular effects of the Cel5A-h38–Cel2-NO pair in a mouse model. (a) Experimental schedule for evaluating the potential systemic effects of the Cel5A-h38–Cel2-NO pair in a mouse model. (b) Cardiac echo measurement was performed at different timepoints, and cardiac function indicators of left ventricular-ejection fraction (LV-EF), left ventricular-fractional shortening (LV-FS), left ventricular internal diameter at end diastole (LVIDd), and left ventricular end-diastolic volume (LV-EDV) were evaluated accordingly. Cel2-NO is catalyzed by Cel5A-h38, Gal-NO is catalyzed by endogenous β-galactosidase. (c) Systolic blood pressure (SBP) was measured daily using a tail cuff in conscious mice. (d-e) Representative ultrasound images (d) and quantification (e) of the relative arterial diameter change ((diastolic diameter - systolic diameter) / systolic diameter) in response to the indicated treatments at different timepoints. (f-g) Representative H&E staining of the heart and artery from mice treated with saline, Cel2-NO, or Gal-NO (1 mg/mL, 100 μL, i.v., every two days), collected at 7 days after initial injection. f, Scale bars, 1 mm (top), 100 μm (bottom). g, Scale bars ,100 μm (top), 50 μm (bottom). n = 3 per group. Two-way ANOVA with Tukey's post-hoc analysis was performed. All data are presented as the means ± SEM. Differences were considered to be significant at $p < 0.05$.

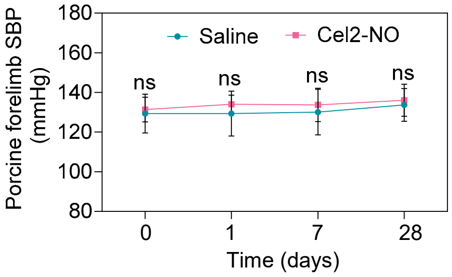

Figure S7. Effects of NO treatment on porcine forelimb systolic blood pressure (SBP). Porcine forelimb SBP was measured before (0 d) and after Cel2-NO administration at indicated time points (1, 7, and 28 d). n =3 per group. All data are presented as the means ± SEM. Differences were considered to be significant at $p < 0.05$.

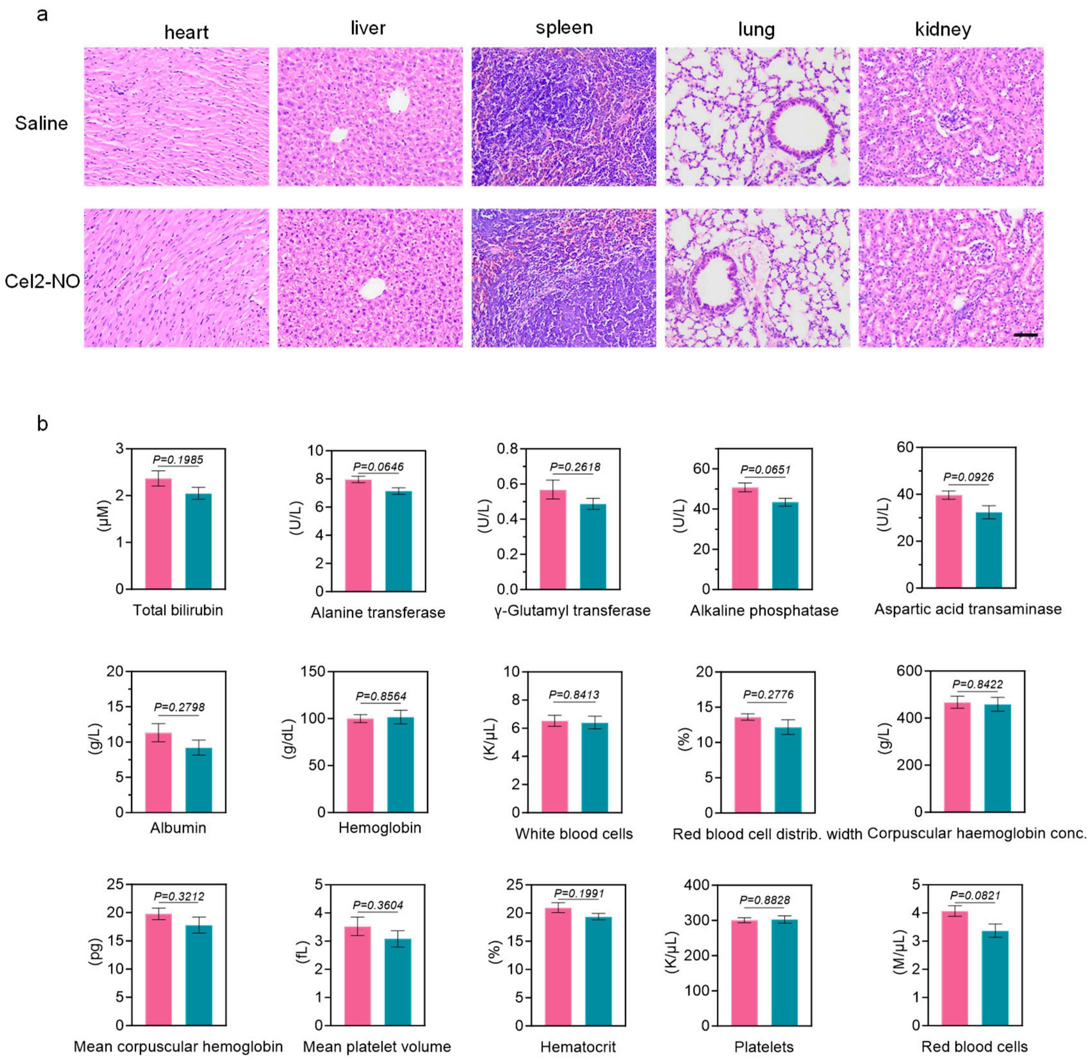

Figure S8. Tissue compatibility of the NO prodrug. (a) Representative H&E staining of heart, liver, spleen, kidney and lung from control and Cel2-NO-treated mice, collected at 7 days after i.v. injection of saline, Cel2-NO (1 mg/mL, 100 μL). Scale bars, 50 μm. (b) Blood biochemistry and blood routine of mice treated with saline (red) and Cel2-NO (green). The blood was collected at 7 days post-injection. n = 3 per group. All data are presented as the means ± SEM. Unpaired two-tailed Student's t test was performed. Differences were considered to be significant at $p < 0.05$.

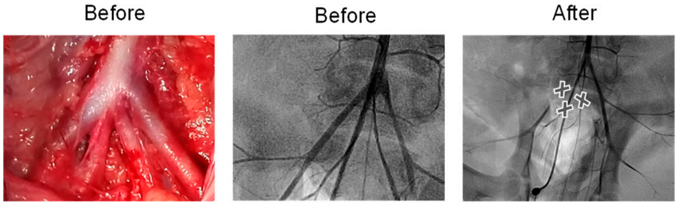

Figure S9. Representative surgical image (pre-ligation) and post-procedure angiogram (after contrast injection in the distal aorta) for the triple ligation model.

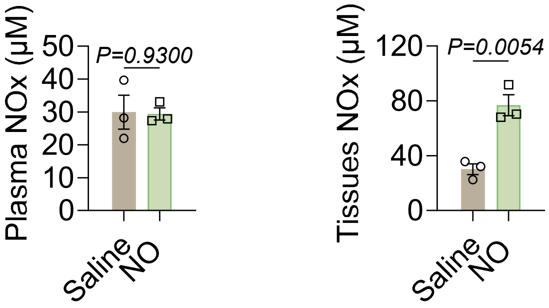

Figure S10. The levels of NO in plasma and tissues at 28 days. All data are presented as the means ± SEM. Unpaired two-tailed Student's t test was performed. Differences were considered to be significant at $p < 0.05$.
